# EmiratesMEDs: a national competency framework for UAE medical graduates

**DOI:** 10.3389/fmed.2026.1803817

**Published:** 2026-06-12

**Authors:** Mohi Magzoub, Mohamed Hassan Taha, Susan Ann Waller, Fouzia Shersad, Emad Nosair, Pankaj Lamba, Senthil Kumar Rajasekaran, Hatem Alameri, Mohammed Al-Houqani, Amjad M. Qandil

**Affiliations:** 1 Department of Medical Education, College of Medicine and Health Sciences, United Arab Emirates University, Al Ain, United Arab Emirates; 2 Medical Education Center and College of Medicine, University of Sharjah, Sharjah, United Arab Emirates; 3 Adjunct Senior Research Fellow, Monash Rural Health’s Rural Nursing & Allied Health, Monash University, Clayton, VIC, Australia; 4 National Institute for Health Specialties, United Arab Emirates University, Al Ain, United Arab Emirates; 5 Department of Basic Medical Sciences, College of Medicine, University of Sharjah, Sharjah, United Arab Emirates; 6 College of Medicine, Gulf Medical University, Ajman, United Arab Emirates; 7 College of Medicine and Health Sciences, Khalifa University, Abu Dhabi, United Arab Emirates; 8 Commission of Academic Accreditation, Abu Dhabi, United Arab Emirates

**Keywords:** competency-based medical education, curriculum alignment, EmiratesMEDs, entrustable professional activities, medical graduate outcomes, national competency framework, UAE healthcare system, undergraduate medical education

## Abstract

**Background:**

Competency-based medical education (CBME) focuses on outcome-driven training that aligns with healthcare system needs. Although national frameworks have enhanced medical education worldwide, the UAE lacked a unified model reflecting its unique context before this framework was introduced. The Emirates Medical Education Directives EmiratesMEDs initiative was created to establish national graduate outcomes, blending global standards with local relevance. This article describes the development, structure, and features of the EmiratesMEDs framework, and its role in harmonizing undergraduate medical education across UAE medical colleges.

**Methods:**

The EmiratesMEDs framework was crafted by a national task force consisting of representatives from all UAE medical colleges, regulators, and academic experts. A multi-phase, mixed-methods approach was used, including: (1) a literature review of global CBME models, (2) consensus workshops to identify thematic roles and core competencies, (3) stakeholder consultations via surveys, focus groups, and interviews, (4) multiple Delphi rounds to refine competencies and entrustable professional activities (EPAs), (5) data analysis and integration, and (6) external expert validation.

**Results:**

The EmiratesMEDs framework includes nine thematic roles, each associated with a core competency and supported by 85 enabling competencies. It also defines 14 EPAs that connect competencies to real clinical tasks expected of graduates, such as conducting patient assessments and managing acute conditions. The framework offers detailed guidance, including clinical presentations and procedural skills, to facilitate curriculum development and assessment.

**Conclusion:**

EmiratesMEDs establishes a unified, context-specific competency framework for UAE medical graduates. It aligns educational outcomes with national health priorities and international standards, providing clear guidance for curriculum development, assessment, and accreditation across UAE medical colleges.

## Background

Competency-Based Medical Education (CBME) has gained international prominence as a framework that emphasizes outcomes, learner development, and alignment with healthcare needs. Rather than relying on time-based progression, CBME promotes the attainment and demonstration of defined competencies before learners advance. This model has been embraced across multiple regions and adapted into national frameworks such as CanMEDS in Canada, ACGME core competencies in the United States, Tomorrow’s Doctors in the United Kingdom, and SaudiMEDs in the Kingdom of Saudi Arabia. These frameworks have contributed to enhancing the consistency of medical training and ensuring that graduates are prepared to meet the demands of modern healthcare practice (1–4). A central rationale behind CBME is its emphasis on preparing physicians not only to provide high-quality clinical care, but also to engage with broader system-level responsibilities. This includes interprofessional collaboration, patient safety, leadership, health advocacy, and responding to the needs of diverse populations (5, 6). In line with the concept of social accountability, medical education is increasingly expected to produce graduates who can address local health priorities while also meeting global standards (7). The World Federation for Medical Education (WFME) and the Lancet Commission on Health Professionals for the 21st Century have both called for competency-based models that align medical education with health system performance and equity goals (8, 9).

In this context, the development of national or regional competency frameworks has emerged as a key strategy for guiding undergraduate medical education, ensuring curriculum alignment, and promoting transparent outcomes. These frameworks articulate shared expectations across institutions and serve as a basis for assessment, accreditation, and continuous improvement (10). When developed through collaborative stakeholder engagement, they also foster trust, coherence, and relevance to societal needs (11).

The United Arab Emirates (UAE) presents a unique setting for implementing such a national framework. Its healthcare system is characterized by rapid modernization, investment in digital health and innovation, and a strong emphasis on international benchmarking. The United Arab Emirates presents a unique healthcare and medical education landscape, shaped by its federal structure of seven emirates and their respective governance arrangements. Within this system, each emirate has its own degree of constitutional autonomy in health and education. Medical colleges are distributed unevenly across the country: Dubai has two medical colleges; Abu Dhabi hosts the oldest and largest medical college; Ajman has two medical colleges; Sharjah has one medical college; and Ras Al Khaimah has one medical college, whereas Fujairah and Umm Al Quwain currently do not have medical colleges. Against this backdrop, the EmiratesMEDs framework provides a timely opportunity to harmonize medical education across the seven emirates. Such harmonization is likely to enhance the quality of medical education through greater collaboration, shared learning, and exchange of experience. The UAE population is highly diverse, composed of multiple nationalities, cultures, and languages, requiring future physicians to be culturally competent and effective communicators. At the same time, the country faces a growing burden of non-communicable diseases, such as diabetes, cardiovascular conditions, and cancer, which are emerging earlier in life and increasing the demand for prevention-oriented care (5, 6).

The UAE has a well-established network of medical colleges that have adopted international best practices and undergone accreditation from national and global bodies. However, until recently, there was no published national undergraduate competency framework that unified expectations across institutions while anchoring them in the specific health priorities and context of the UAE. This created an opportunity to design a shared framework that would consolidate existing strengths, promote alignment with the UAE Qualifications Framework (QF-Emirates), and further advance the goals of medical education across the country.

The EmiratesMEDs initiative was launched to address this opportunity. Drawing inspiration from successful international models and guided by the principles of relevance, social accountability, and global comparability, the EmiratesMEDs framework seeks to articulate the essential roles, competencies, and expected outcomes of medical graduates in the UAE. This paper presents the conceptual foundation of EmiratesMEDs, situates it within the global movement toward CBME, and outlines the rationale for a nationally shared framework rooted in the UAE’s distinct healthcare landscape.

## Methodology for developing the framework

The EmiratesMEDs framework was developed through a structured, collaborative, and iterative process led by the National Competency Taskforce using a mixed method approach. The EmiratesMEDs framework was developed using a multi-phase, mixed-methods approach, combining both qualitative and quantitative strategies. This approach was chosen to ensure that the framework was grounded in evidence, informed by stakeholder perspectives, and aligned with both national priorities and international standards. Similar approaches, particularly those incorporating literature review and Delphi-based consensus, are widely used in competency framework development to enhance validity and acceptability. The National Competency Taskforce was established in February 2021 through a partnership between the Deans of all accredited medical colleges in the United Arab Emirates (UAE), the Commission for Academic Accreditation (CAA) under the Ministry of Education, and the National Institute for Health Specialties (NIHS). The task force included representatives from every medical school in the UAE, alongside experts from regulatory, academic, and healthcare institutions, including the UAE Qualifications Framework (QF-Emirates), health service authorities, and academic leaders in medical education.

The taskforce was established with the aim of: Agreeing on the overarching themes of the framework, defining competencies and program learning outcomes (CLOs/PLOs), identifying enabling competencies and sub-competencies, developing and a list of Entrustable Professional Activities (EPAs) establishing strategies for dissemination and implementation

The development process followed a mixed-methods design consisting of five main phases:

### Phase I: needs analysis, benchmarking, and initial drafting

This initial phase involved a scoping review of global and regional competency-based medical education (CBME) frameworks. The aim was to identify internationally recognized structures and methods used in defining physician competencies. Key references included CanMEDS (Canada) (1), ACGME Core Competencies (USA) (2), Tomorrow’s Doctors (UK) (3), SaudiMEDs (Saudi Arabia) (4), AAMC Physician Competency Reference Set (USA) (12), and the Scottish Doctor framework (13). These models informed the initial thematic structure of EmiratesMEDs, particularly the definition of competency domains, role-based structuring, and the use of Entrustable Professional Activities (EPAs) (10) to enhance assessment and integration across competencies, two specialized sub-taskforces were formed: one tasked with drafting enabling competencies aligned with each role, and the second with developing a set of context-relevant EPAs. Each sub-taskforce independently conducted literature reviews, needs assessments, and benchmarking exercises, leading to the development of initial drafts of competencies, EPAs, and clinical skills.

### Phase II: stakeholder engagement and consensus building

Between 2021 and 2022, extensive stakeholder consultation was undertaken using a combination of quantitative and qualitative methods. A web-based survey was developed and distributed to stakeholders across UAE medical colleges, including educators, healthcare providers, accreditation bodies, employers, and graduates. The survey included Likert-scale items and open-ended questions to assess the relevance, clarity, and applicability of proposed competencies and EPAs. In parallel, 13 focus group discussions involving more than 170 participants were conducted to explore key themes, contextual relevance, and implementation considerations. Semi-structured interviews and national workshops further enriched stakeholder input. Findings from these activities informed a modified Delphi process, through which structured feedback was obtained iteratively from stakeholders. An additional 40 participants contributed through survey feedback. The Delphi process in this phase was conducted over two iterative rounds, with participants reviewing and rating competencies and EPAs for relevance, clarity, and applicability. Consensus was predefined as ≥ 80% agreement among participants. Items not meeting this threshold were revised and redistributed for further evaluation. Anonymity of responses was maintained during Delphi rounds to minimize dominance bias and ensure independent judgment. This phase resulted in refinement of the competency framework and the identification of enabling competencies, clinical presentations, clinical skills, and EPAs.

### Phase III: alignment, mapping, and refinement

During the iterative phase from 2022 to 2023, further refinement was undertaken through Delphi rounds, mapping exercises, and consensus meetings involving 14 experts representing key stakeholder groups. Competencies were systematically aligned with EPAs, assessment strategies, and international frameworks. The sub-taskforces defined levels of achievement, milestones across stages of training, and behavioral descriptors. A total of two additional Delphi rounds were conducted in this phase to finalize competencies and EPAs. The same consensus threshold ( ≥ 80% agreement) was applied. Items failing to achieve consensus after two rounds were discussed in structured consensus meetings and resolved through majority agreement. This process led to the development of a semi-final framework with clearly structured competencies, EPAs, and supporting elements.

### Phase IV: external validation

In 2023, the near-final framework underwent external validation through consultation with five international experts, including representatives from ACGME, SaudiMEDS, and FAIMER. These experts reviewed the framework for clarity, coherence, and alignment with international standards. Their feedback informed final refinements and confirmed the content and face validity of the framework.

### Phase V: implementation and dissemination

Following validation, the framework entered the implementation phase in 2023. Engagement activities included national workshops, consultations, and faculty meetings involving approximately 600 faculty members and stakeholders across UAE medical colleges. This phase focused on dissemination, capacity building, and gathering feedback to support adoption. Feedback obtained during this stage informed further refinement of the framework and preparation for national implementation.

**FIGURE 1:**
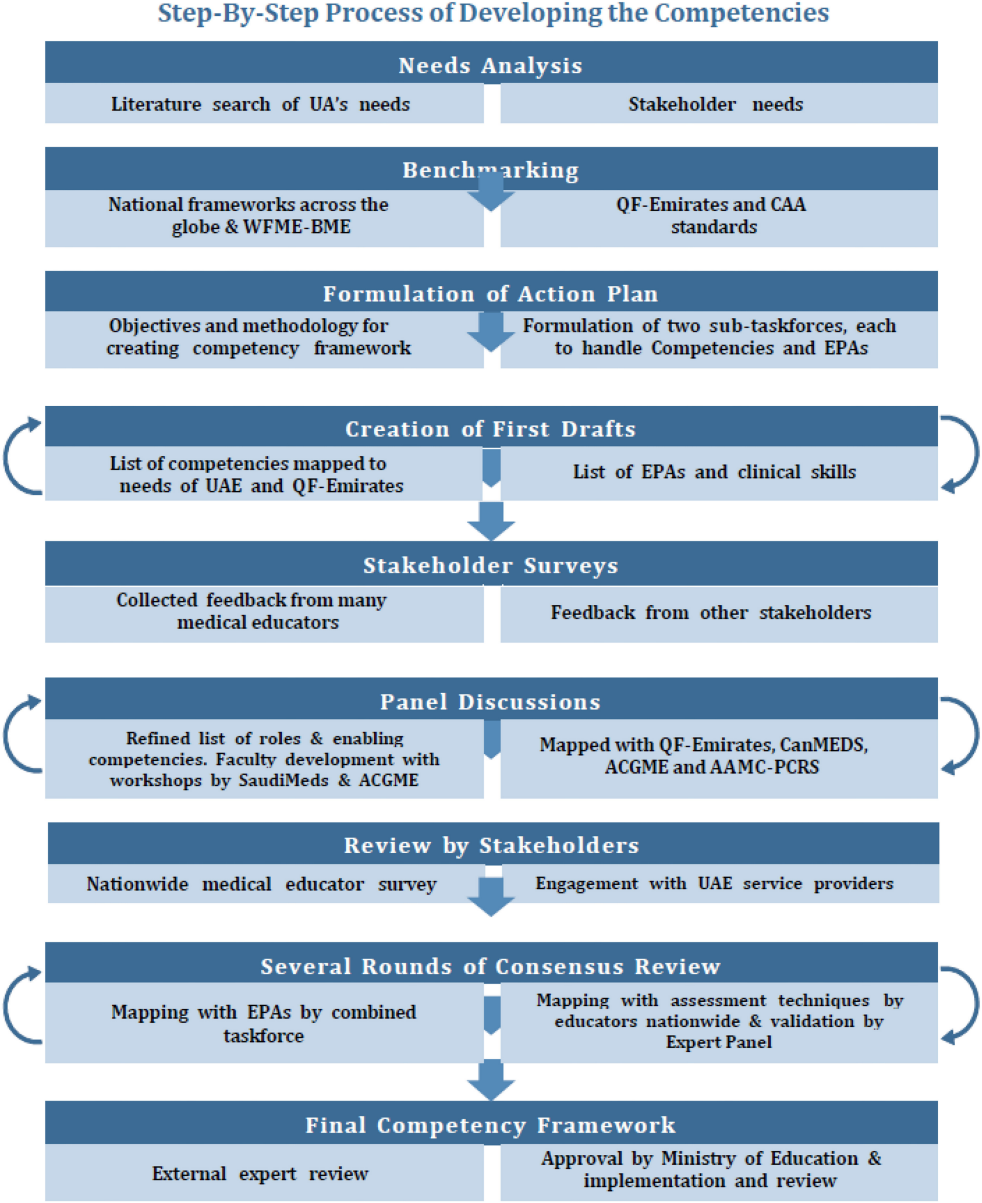
Steps of the development of the EmiratesMEDs competency framework. Source: EmiratesMEDs framework: https://www.caa.ae/PORTAL GUIDELINES/2023-04-27%20UAE%20Competency%20Framework%20For%20Medical%20Education,%20EmiratesMeds.pdf.

Figure 1 provides a summary of the EmiratesMEDs development process.

**FIGURE 2:**
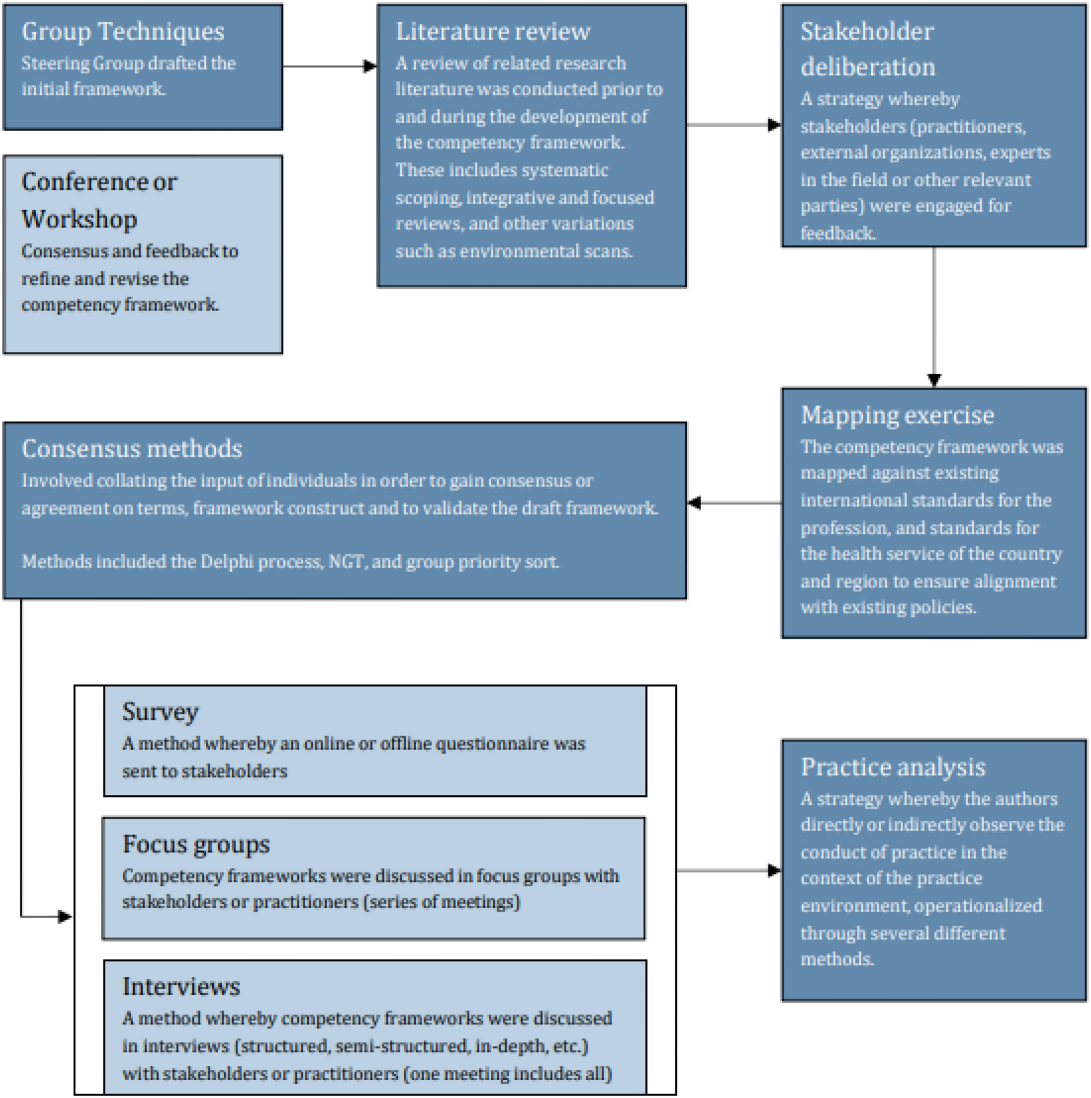
Summary of EmiratesMEDs competency framework. Source: EmiratesMEDs framework: https://www.caa.ae/PORTALGUIDELINES/2023-04-27% 20UAE%20Competency%20Framework%20For%20Medical%20Education,%20EmiratesMeds.pdf.

Figure 2 outlines the stepwise methodological phases that guided framework creation, validation, and endorsement. The phases are summarized in Table 1.

**TABLE 1:** Phrases, instruments, number of participants and outcomes.

Phase	Date	Instruments	Number of participants	Outcome
Phase I Needs analysis, benchmarking and initial drafting	2021 early phase	Literature review, benchmarking and needs analysis	Ten representatives formed the national taskforce (including representatives from all UAE medical colleges, Commission of Academic accreditation, QF-Emirates and health authorities) reviewed the literature Searching Medline, Scopus, Web of Sciences, CINAHL and PsycINFO Two Sub-taskforces (competency group and EPA/clinical skills group) with expert members from the national taskforce	Based on international similar frameworks and national needs a preliminary list of competencies were identified
Phase II Stakeholder Engagement and Consensus Building	2021 to 2022	Focus group discussions, *workshops, surveys, semi-structured interviews, Delphi rounds*, Web-based Survey	13 Focus group Discussions (FGD) including 170 participants representing academia, health system leaders, healthcare providers, accreditation bodies, and graduates Obtained feedback from 40 participants representing the above groups	Refinement of the competencies list Identifying preliminary list of enabling competencies, clinical presentation and clinical skills in addition to EPAs
Phase III Alignment, mapping and refinement	Iterative phase 2022-2023	Delphi methods Mapping exercises, consensus meetings	14 participants representing experts from different stakeholders	Semi-final framework with aligned competencies, EPAs, milestones, and behavioral descriptors
Phase IV External validation	Final phase before approval in 2023	Interview with experts from ACGME, SaudiMEDS, FAIMER USA,	5 global experts including ACGME, SaudiMEDS, FAIMER)	Final validated Framework aligned with international standards
Phase V implementation and dissemination	Post-validation in 2023	Faculty meeting including workshops consultations and faculty engagement and *World Café methodology in a national conference held in 2023*	600 Faculty members across UAE medical colleges and stakeholders involved in implementation	Update the framework based on feedback Framework refinement, dissemination, and preparation for national adoption

### Ethics consideration

The project is approved by the Social Sciences Ethics Committee at the United Arab Emirates University, research number: ERSC_2024_5464. Informed consent to participate was obtained from all participants prior to data collection. The study was conducted in accordance with the principles of the Declaration of Helsinki.

## Results

Table 1 Summarized data on the different phases of the framework development, including overall responses received and how many from each category. The table presents as well as the main modifications after each phase.

The EmiratesMEDs framework provides a national, comprehensive competency-based framework designed to guide undergraduate medical education in the United Arab Emirates. It offers a structured approach to aligning graduate outcomes with national health priorities, international standards, and the practical demands of clinical practice. The framework comprises four integrated components: thematic roles, core competencies, enabling competencies, and entrustable professional activities (EPAs). These are further supported by detailed lists of clinical presentations and procedural skills intended to assist curriculum development and implementation.

**FIGURE 3:**
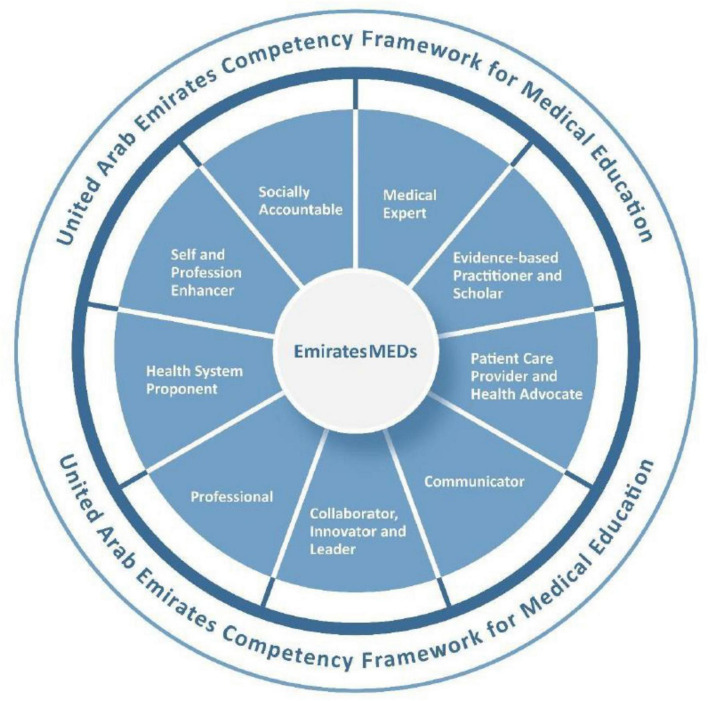
Thematic roles of the competency framework for medical education in the United Arab Emirates-EmiratesMEDs. Source: EmiratesMEDs framework: https://www.caa.ae/PORTALGUIDELINES/2023-04-27%20UAE%20Competency%20Framework%20For%20Medical%20Education,%20EmiratesMeds.pdf.

At the core of the framework are nine thematic roles, each representing a major domain of medical practice Figure 3. These roles define the broad expectations of physicians within the UAE health system and were established through national consultation and consensus. For each thematic role, a corresponding core competency was identified, reflecting the key function and expected performance related to that domain. Together, these nine core competencies outline the essential attributes and skills that every medical graduate in the UAE should demonstrate upon graduation.

The Supplementary Annex 1 presents the 85 enabling competencies with the mapped EPAs and Clinical skills.

The thematic roles span clinical expertise, scholarly practice, population-focused care, communication, collaboration, professionalism, systems-based thinking, personal development, and social accountability. The “Medical Expert” role emphasizes the mastery of clinical knowledge and skills that form the bedrock of medical practice. The “Evidence-Based Practitioner and Scholar” role highlights the importance of applying scientific reasoning and continuous learning to improve patient care. The “Patient Care Provider” and “Health Advocate” role calls for responsiveness to the health needs of both individuals and communities. The “Communicator” role emphasizes the importance of clear, empathetic, and effective communication with patients, their families, and healthcare teams. The “Collaborator, Innovator, and Leader” role reflects the evolving demand for teamwork, adaptive thinking, and leadership in complex healthcare environments. The “Professional” role affirms commitment to ethical practice, integrity, and accountability. The “System-Based Healthcare Advocate” role prepares graduates to understand and influence health system structures to promote better care. The “Self and Profession Enhancer” role addresses the importance of lifelong learning, wellness, and contribution to the profession. Finally, the “Socially Accountable Practitioner” role captures the UAE’s commitment to ensuring that medical graduates are prepared to serve diverse populations and respond to pressing societal health needs.

To translate these thematic roles into measurable educational outcomes, the EmiratesMEDs framework defines a total of 85 enabling competencies (see Supplementary Appendices). These competencies offer specific, observable behaviors that support the realization of each core competency. They cover a wide range of domains including clinical decision-making, ethical reasoning, patient safety, health advocacy, quality improvement, research literacy, teamwork, and leadership. The formulation of these enabling competencies was informed by global benchmarking, national consultation, and iterative refinement through the Delphi method. They are designed to guide curriculum development, shape assessment strategies, and facilitate student feedback.

In addition to defining what graduates should know and be able to do, the EmiratesMEDs framework incorporates 14 Entrustable Professional Activities. These EPAs represent authentic clinical tasks that a medical graduate should be able to perform independently upon entering residency. Each EPA links competencies to practice by identifying the knowledge, skills, and attitudes needed for safe, unsupervised performance. The EPAs include activities such as conducting a comprehensive patient history and physical examination, interpreting diagnostic tests, managing acute and chronic conditions, performing selected procedures, collaborating with interprofessional teams, and ensuring safe transitions of care. These activities provide a structured basis for workplace-based assessments and are meant to serve as bridges between undergraduate training and postgraduate clinical responsibilities. The full list of EPAs is provided in Box 1.

BOX 1 **Entrustable professional activities of EmiratesMEDs.**

**EPA 1:** Obtain, perform, and document a focused or comprehensive history and physical examination that identifies relevant clinical findings.

**EPA 2:** Synthesize information from a clinical encounter to generate and prioritize a justified differential diagnosis.

**EPA 3:** Select, request, and interpret common diagnostic and screening tests to answer clinical questions and inform patient care.

**EPA 4:** Formulate, communicate, and implement a patient-centered management plan, including follow-up and escalation when needed.

**EPA 5:** Accurately document a clinical encounter in the patient record, including relevant findings, assessment, and plan.

**EPA 6:** Deliver a concise, organized oral presentation of a clinical encounter that includes pertinent findings, clinical reasoning, and management plan.

**EPA 7:** Formulate answerable clinical questions, retrieve and appraise relevant evidence, and apply it to advance patient care.

**EPA 8:** Give and receive patient handovers using a structured approach that ensures safe transfer of responsibility and accountability for care.

**EPA 9:** Collaborate effectively with members of an interprofessional team to develop, communicate, and document a shared patient care plan.

**EPA 10:** Recognize patients and situations requiring urgent or emergent care, initiate immediate evaluation and management, and escalate appropriately.

**EPA 11:** Obtain and document informed consent for tests or procedures by explaining indications, benefits, risks, alternatives, and confirming patient understanding.

**EPA 12:** Safely perform general physician procedures appropriate to the local context, with appropriate preparation, technique, aftercare, and documentation.

**EPA 13:** Identify system failures, report patient safety concerns, and contribute to quality improvement actions that promote a culture of safety.

**EPA 14:** Educate patients and families and promote the health of the community through counseling, prevention, and health promotion activities.

The framework is further detailed through two extensive appendices. Supplementary Appendix 1 provides a comprehensive list of clinical presentations that graduates should be prepared to recognize and manage. These presentations are organized across systems and include both common and high-stakes conditions. This list ensures that undergraduate medical education remains grounded in patient-centered care and prepares learners for real-world encounters. The framework further details 70 essential procedural skills, of which 26 are diagnostic procedural skills and 44 therapeutic procedural skills (see Supplementary Appendices). These skills are grouped into six major domains: basic practical procedures, communication and cognitive skills, general and system-specific clinical examinations, diagnostic procedures, assessment procedures, and therapeutic interventions. These are intended to support curriculum mapping, simulation-based education, and the design of performance-based assessments such as Objective Structured Clinical Examinations (OSCEs).

## Discussion

The development and implementation of the EmiratesMEDs framework marks a pivotal milestone in aligning undergraduate medical education in the United Arab Emirates (UAE) with both the country’s evolving healthcare landscape and international best practices. As a national initiative, EmiratesMEDs reflects a deliberate and inclusive effort to contextualize competency-based medical education (CBME), addressing the pressing need for frameworks that are locally relevant, socially accountable, and future-oriented. A key strength of the EmiratesMEDs framework lies in its structured yet flexible architecture, which cascades from thematic roles to core and enabling competencies, then maps onto clinical presentations, procedural skills, and EPAs. This coherent layering facilitates curriculum alignment, standardizes expected outcomes across institutions, and supports both formative and summative assessment strategies. The inclusion of clinical conditions and tasks offers operational clarity for educators and accreditation bodies, contributing to transparent benchmarking of student performance.

The integration of 14 EPAs represents a significant advancement in linking theoretical competence to authentic clinical performance. These EPAs serve as observable and assessable units of professional practice that define readiness for workplace responsibility. As emphasized by ten Cate and others, EPAs operationalize the abstract nature of competencies and bridge the gap between curriculum and clinical care (10). The EmiratesMEDs framework explicitly maps each EPA to relevant enabling competencies, providing a practical roadmap for implementation across diverse learning environments. The approach aligns with international trends toward workplace-based assessment, mirroring initiatives in frameworks such as CanMEDS and the AAMC Core EPAs. What sets EmiratesMEDs apart, however, is its deep embedding in the UAE’s healthcare context. The UAE’s rapid population growth, multicultural society, high burden of non-communicable diseases, and ambitious health system reforms require a generation of physicians capable of not only delivering care but also leading system transformation. Accordingly, EmiratesMEDs incorporates roles such as “System-Based Healthcare Advocate” and “Patient Care Provider” and “Health Advocate,” which resonate strongly with national policy directions.

The UAE’s unique structure as a federation of emirates and its centralized educational governance allowed for coordinated national engagement. All accredited medical colleges, healthcare regulators, and relevant ministries were actively involved in the iterative development process. This comprehensive engagement is consistent with literature advocating for whole-system involvement in CBME reform. Unlike decentralized models used in countries like the United States or Canada, the UAE’s central structure enabled consensus-building and rapid policy alignment. The result is a competency framework that is both nationally unified and adaptable to local institutional contexts.

Social accountability is another hallmark of the EmiratesMEDs framework. The principle, first articulated by Boelen and colleagues, posits that medical colleges should align their education, research, and service with the priority health needs of the communities they serve (14). In EmiratesMEDs, social accountability is not only defined as one of the nine thematic roles but also systematically integrated across enabling competencies and EPAs. This reinforces the UAE’s national vision of promoting equity, preventative care, and health advocacy. A comparative lens with other national frameworks reveals additional distinctions. The AAMC Physician Competency Reference Set and CanMEDS emphasize roles like scholar, communicator, and professional (1, 12), but EmiratesMEDs expands on these with its own emphasis on innovation, leadership, population health, and system-based thinking.

The methodological rigor underpinning EmiratesMEDs further strengthens its credibility. The initiative combined a scoping literature review, national web-based surveys, key informant interviews, focus groups, and a structured modified Delphi process to achieve consensus. The Delphi technique is particularly suited for building agreement among experts from varied backgrounds and is widely endorsed in medical education research. This iterative approach allowed for prioritization, refinement, and validation of competencies. The process was additionally bolstered by validation from an external expert advisory panel, which enhanced objectivity and assured international quality standards.

Reflections on the development process yield several valuable lessons. First, meaningful stakeholder engagement from the outset especially involving faculty, deans, regulators, and students was crucial for achieving national ownership and ensuring future implementation success. Second, grounding the framework in national health priorities and regulatory expectations helped ensure relevance and institutional alignment. Third, the layered structure of the framework supports longitudinal curriculum mapping and assessment planning, addressing a commonly cited challenge in CBME implementation (15).

However, implementation remains the most complex phase. Faculty development, student orientation, and institutional readiness are essential for translating the framework into tangible change in teaching and learning. Existing studies suggest that even well-designed frameworks can falter if educators are not adequately supported or if assessment strategies are misaligned (16). Thus, future phases of EmiratesMEDs should focus on developing faculty toolkits, assessment blueprints, and national monitoring indicators.

A study from one medical school in the UAE reported that the implementation of the Unified Competency Framework for Medical Education (UCFME) represented a transformative step in aligning undergraduate medical education with international best practices, enhancing consistency, and supporting competency-based assessment across curricula (17). This experience resonates with the EmiratesMEDs initiative, which was developed to establish a nationally unified framework reflecting the UAE’s unique health system priorities. Both frameworks emphasize structured change management, faculty engagement, and robust assessment strategies to embed CBME in practice. The EmiratesMEDs framework makes a significant contribution to the global discourse on national medical education reform. It builds on and goes beyond previous models by offering a blueprint for countries with centralized education systems seeking to embed CBME and EPAs in a context-sensitive, socially accountable manner. The integration of population health, system-based care, and health advocacy provides a forward-looking vision of the physician’s role in the 21st-century Arab world. Further research is needed to evaluate the implementation and impact of EmiratesMEDs. Key areas include student competency progression, graduate career trajectories, and alignment of institutional curricula with national health outcomes. Such evaluation will not only inform future iterations of the framework but also contribute to global understanding of effective CBME implementation in rapidly changing health systems. There are some limitations associated with the development of this framework. Although the process involved broad national engagement, including representatives from all accredited medical colleges and regulatory bodies, the voices of students and patients were underrepresented in the later stages of decision-making.

While the Delphi method supported structured consensus-building, there remains a possibility that certain competencies reflect stronger input from more dominant institutions, potentially limiting balanced representation. Further studies are needed to explore implementation experiences, assess educational and system-level outcomes, and guide iterative refinement of the framework.
