# Supplementary material for: EmiratesMEDs: a national competency framework for UAE medical graduates
Source: Front Med (Lausanne). 2026 Jun 12;13:1803817. doi: 10.3389/fmed.2026.1803817 (PMC13303357; doi:10.3389/fmed.2026.1803817)
Supplement: Supplementary file 1 [file Data_Sheet_1.docx]

|  |
| --- |

**Annex 1**

**Thematic roles, Core Competencies, & Enabling Competencies**

| **Thematic Role** | **Core Competency** | **Comp. code** | **Enabling Competencies** | **EPA** |
| --- | --- | --- | --- | --- |
| **Theme 1: Medical Expert** | **Demonstrate knowledge of established and evolving biomedical, clinical, epidemiological, and psychosocial sciences, and apply this knowledge to patient and population care through the clinical reasoning approach.** | ME1 | Demonstrate an investigatory and analytic approach to clinical situations | EPA 2, 3, 6, 7 |
|  |  | ME2 | Apply established and emerging biophysical scientific principles to health care for patients and populations | EPA 1, 2, 3, 4, 6, 7, 13, 14 |
|  |  | ME3 | Apply established and emerging principles of clinical sciences to diagnostic and therapeutic decision-making, clinical problem solving, and other  aspects of evidence-based health care. | EPA 2, 3, 4, 7, 12 |
|  |  | ME4 | Apply principles of epidemiological sciences to the identification of health problems, risk factors, treatment strategies, resources, health system development, and disease prevention/health promotion efforts for patient  and population care. | EPA 13, 14 |
|  |  | ME5 | Apply principles of social-behavioral sciences to the provision of patient- centered and relationship-based care, including assessment of impact of psychosocial-cultural influences on health, disease, care-seeking, care compliance, as well as barriers to and attitudes toward patient and population care | EPA 1, 4, 14 |
|  |  | ME6 | Contribute to the creation, dissemination, application, and translation of new health care knowledge and practices | EPA 7, 13 |
|  |  | ME7 | Demonstrate an ability to manage infodemic (too much information including false or misleading information in digital and physical environments during a disease outbreak) while respecting cultures, social values, and legislation in UAE. | EPA 13, 14 |

| **Thematic Role** | **Core Competency** | **Comp. code** | **Enabling Competencies** |  |
| --- | --- | --- | --- | --- |
| **Theme 2: Evidence-based Practitioner and Scholar** | **Demonstrate ability to continuously improve through self- evaluation and lifelong learning; and ability to investigate and evaluate patient care using an evidence- based approach gathered from high quality research, practitioner experience, and based on value to the patient, while considering the context of care.** | EBS1 | Systematically analyze practice using quality improvement methods and implement changes with the goal of practice improvement. | EPA 7, 13, 14 |
|  |  | EBS2 | Incorporate feedback into daily practice. | EPA 7 |
|  |  | EBS3 | Inquire, gather, assess, and apply evidence from scientific studies related to  patients’ health problems. | EPA 3, 5, 7 |
|  |  | EBS4 | Demonstrate use of information technology systems and health informatics to support evidence-based care. | EPA 4, 7, 13 |
|  |  | EBS5 | Obtain and utilize information about individual patients, populations of patients, or patients’ communities to improve care. | EPA 2, 13, 14 |
|  |  | EBS6 | Continually identify, analyze, and implement new knowledge, guidelines, standards, technologies, products, and services to improve outcomes. | EPA 3, 5, 7 |
|  |  | EBS7 | Demonstrate knowledge management and translation ability to improve patient and population care and the healthcare system as a whole. | EPA 7, 13, 14 |
|  |  | EBS8 | Demonstrate ability to conduct research following scientific approach, including writing a proposal, implementing research, manuscript writing, and disseminating findings guided by research ethics. | EPA 7, 13 |

| **Thematic Role** | **Core Competency** | **Comp. code** | **Enabling Competencies** |  |
| --- | --- | --- | --- | --- |
| **Theme 3: Patient Care Provider and Health Advocate** | **Provide patient-and population-centered care that is compassionate, appropriate, and effective for the management and prevention of common health problems; and advocate patient rights and patient safety.** | PC1 | Perform all medical, diagnostic, and surgical procedures considered  essential for the area of practice as a generalist. | EPA 6 |
|  |  | PC2 | Gather essential and accurate information about patients and their  conditions through a clinical reasoning approach involving history-taking, physical examination, and the use of laboratory data, imaging, and other tests. | EPA 1, 2 |
|  |  | PC3 | Organize and prioritize responsibilities to provide safe, effective, and  efficient care. | EPA 7 |
|  |  | PC4 | Interpret laboratory data, imaging studies, and other tests required for the  area of practice as a generalist. | EPA 2, 3 |
|  |  | PC5 | Make informed decisions about diagnostic and therapeutic interventions  based on patient information and preferences, up-to-date scientific evidence, and clinical judgment. | EPA 3, 5 |
|  |  | PC6 | Develop and conduct effective and appropriate patient management plans. | EPA 5 |
|  |  | PC7 | Counsel and educate patients and their families to empower them to participate in their care and enable shared decision-making when appropriate. | EPA 4 |
|  |  | PC8 | Provide appropriate referral of patients including ensuring continuity of  care throughout transitions between providers or settings and following up on patient progress and outcomes. | EPA 7, 12 |
|  |  | PC9 | Provide health care services to patients, families, and communities to prevent health problems or maintain health. | EPA 13, 14 |
|  |  | PC10 | Advocate for health through appropriate health promotion strategies and interventions grounded on social determinants of health. | EPA 13, 14 |
|  |  | PC11 | Participate in the education of patients, families, students, trainees, peers,  and other health professionals. | EPA 4, 7 |

| **Thematic Role** | **Core Competency** | **Comp. code** | **Enabling Competencies** |  |
| --- | --- | --- | --- | --- |
| **Theme 4: Communicator** | **Demonstrate written, verbal, and non-verbal interpersonal communication skills that result in effective interactions with patients, their families, and health professionals** | C1 | Communicate effectively with patients, families, and the public, as appropriate, across a broad range of socioeconomic and cultural backgrounds. | EPA 1, 4 |
|  |  | C2 | Demonstrate effective therapeutic relationships with patients and their families that facilitate the gathering and sharing of essential information and develop plans that reflect the patient’s health needs and goals. | EPA 1, 4 |
|  |  | C3 | Communicate effectively with colleagues within one’s profession or specialty, other health professionals, and health-related agencies. | EPA 7 |
|  |  | C4 | Work effectively with others as a member or leader of a health care team or other professional groups. | EPA 7 |
|  |  | C5 | Demonstrate ability to act in a consultative role to other health  professionals. | EPA 12 |
|  |  | C6 | Document and share comprehensive, timely and relevant written and electronic information about medical encounters to optimize clinical decision-making, patient safety, confidentiality, and privacy. | EPA 7 |
|  |  | C7 | Demonstrate sensitivity, honesty, and compassion in difficult conversations (e.g., about issues-such as death, end-of-life issues, adverse events, bad news, disclosure of errors, and other sensitive topics). | EPA 4 |
|  |  | C8 | Demonstrate insight and understanding about emotions and human responses to emotions that allow one to develop and manage interpersonal interactions. | EPA 4 |
|  |  | C9 | Demonstrate the ability to prevent and resolve inter-professional team  conflicts. | EPA 7 |

| **Thematic Role** | **Core Competency** | **Comp. code** | **Enabling Competencies** |  |
| --- | --- | --- | --- | --- |
| **Theme 5: Collaborator, Innovator & Leader** | **Demonstrate the ability to collaborate and lead a health team and actively engage in an interprofessional health team in a manner that optimizes safe, effective patient and population- centered care** | CIL1 | Work with other health professionals to establish and maintain a climate of mutual respect, dignity, diversity, ethical integrity, and trust. | EPA 7 |
|  |  | CIL2 | Use the knowledge of one’s own role and those of other professions to appropriately assess and address the health care needs of the patients and populations served. | EPA 7 |
|  |  | CIL3 | Communicate with other health professionals in a responsive and responsible manner that supports the maintenance of health and the treatment of disease in individual patients and populations. | EPA 7 |
|  |  | CIL4 | Participate in different team roles to establish, develop, and continuously enhance interprofessional teams to provide patient- and population- centered care that is safe, timely, efficient, effective, and equitable. | EPA 7 |
|  |  | CIL5 | Demonstrate leadership skills that enhance team functioning, the learning environment, and/or the health care delivery system. | EPA 7, 13 |
|  |  | CIL6 | Propose creative and innovative initiatives and solutions to priority health problems. | EPA 13, 14 |
|  |  | CIL7 | Promote entrepreneurship and sustainability as cost effective strategies to meet the health needs of the population in UAE. | EPA 13, 14 |

| **Thematic Role** | **Core Competency** | **Comp. code** | **Enabling Competencies** |  |
| --- | --- | --- | --- | --- |
| **Theme 6: Professional** | **Carry out professional responsibilities and activities through demonstration of commitment and adherence to ethical principles** | P1 | Demonstrate compassion, integrity, and respect for others. | EPA 1, 4 |
|  |  | P2 | Demonstrate responsiveness to patient needs that supersede self-interest. | EPA 1 |
|  |  | P3 | Demonstrate respect for patient privacy and autonomy. | EPA 4 |
|  |  | P4 | Demonstrate accountability to patients, society, and the profession as role  models. | EPA 7 |
|  |  | P5 | Demonstrate sensitivity and responsiveness to a diverse patient population in UAE, including but not limited to diversity in gender, age, culture, race, religion, and disabilities. | EPA 1, 14 |
|  |  | P6 | Demonstrate a commitment to ethical principles pertaining to the provision of care, confidentiality, informed consent, and business practices, including  compliance with relevant national laws, policies, and regulations. | EPA 7 |
|  |  | P7 | Exhibit professional behaviors in the use of technology-enabled  communication including social media. | EPA 7 |
|  |  | P8 | Recognize and respond to unprofessional and unethical behaviors in  physicians and other colleagues in health care professions. | EPA 7 |
|  |  | P9 | Recognize and manage conflict of interest. | EPA 7 |
|  |  | P10 | Exhibit self-awareness and manage influences on personal well-being and  Professional performance. | EPA 7 |
|  |  | P11 | Manage personal and professional demands for a sustainable practice throughout the physician life cycle. | EPA 7 |
|  |  | P12 | Promote a culture that recognizes, supports, and responds effectively to colleagues in need. | EPA 7 |
|  |  | P13 | Develop a professional identity acknowledging a commitment to the health and well-being of patients, families, society, and peers. | EPA 7 |

| **Thematic Role** | **Core Competency** | **Comp. code** | **Enabling Competencies** |  |
| --- | --- | --- | --- | --- |
| **Theme 7: System-Based Healthcare Advocate** | **Demonstrate an awareness of and responsiveness to the larger contextand system of health care in UAE, as well as use resources effectively to contribute to the development of the system to provide optimal health care** | HS1 | Work effectively in various health care delivery settings and systems relevant to one’s clinical specialty. | EPA 7 |
|  |  | HS2 | Coordinate patient care within the health care system relevant to one’s clinical specialty. | EPA 7 |
|  |  | HS3 | Incorporate considerations of cost awareness and the risk-benefit analysis of patients and/or population-based care. | EPA 7 |
|  |  | HS4 | Advocate for quality patient care and optimal patient care systems. | EPA 7 |
|  |  | HS5 | Participate in identifying system errors and implementing potential systems solutions. | EPA 7 |
|  |  | HS6 | Perform and practice administrative and management responsibilities commensurate with one’s role, abilities, and qualifications. | EPA 1, 7 |
|  |  | HS7 | Utilize technology and systems responsibly and effectively, maintaining security, ensuring currency, and proposing improvement. | EPA 2, 3 |
|  |  | HS8 | Devise and contribute to developing innovative approaches to improve access and quality of care. | EPA 7 |
|  |  | HS9 | Describe national health care systems, including their organizations, financing, health insurance, policies, and procedures. | EPA 7 |

| **Thematic Role** | **Core Competency** | **Comp. code** | **Enabling Competencies** |  |
| --- | --- | --- | --- | --- |
| **Theme 8: Self and Profession Enhancer** | **Demonstrate the qualities required to sustain lifelong learning and professional growth** | SPE1 | Develop the ability to use self-awareness of knowledge, skills, and emotional wellbeing to engage in appropriate help-seeking behaviors. | EPA 7 |
|  |  | SPE2 | Demonstrate resilience and healthy coping mechanisms to respond to stress. | EPA 7 |
|  |  | SPE3 | Manage conflict between personal and professional responsibilities. | EPA 7 |
|  |  | SPE4 | Practice flexibility and maturity in adjusting to change with the capacity to alter behavior. | EPA 7 |
|  |  | SPE5 | Demonstrate trustworthiness that makes colleagues feel secure when one is responsible for the care of patients. | EPA 7 |
|  |  | SPE6 | Demonstrate self-confidence that puts patients, families, and health-care team members at ease. | EPA 1, 7 |
|  |  | SPE7 | Recognize that ambiguity is part of clinical health care and respond by using appropriate resources in dealing with uncertainty. | EPA 2, 3 |
|  |  | SPE 8 | Identify strengths, deficiencies, and limits in one’s knowledge and expertise. | EPA 7 |
|  |  | SPE 9 | Set learning and improvement goals. | EPA 7 |
|  |  | SPE10 | Identify and perform learning activities that address one’s gaps in knowledge, skills, or attitudes. | EPA 7 |
|  |  | SPE 11 | Manage personal and professional demands for a sustainable practice throughout the physician life cycle; and promote a culture that recognizes, supports, and responds effectively to colleagues in need. | EPA 7 |

| **Thematic Role** | **Core Competency** | **Comp. code** | **Enabling Competencies** |  |
| --- | --- | --- | --- | --- |
| **Theme 9: Socially accountable** | **Meet the health needs of patients and society, demonstrate improved health outcomes, and promote health equity, relevance, collaboration, cost- effectiveness, and quality** | SA1 | Demonstrate an understanding of the influence and potential implications of social determinants on health-related beliefs, behaviors, and outcomes, and incorporate this knowledge into patient care. | EPA 1, 14 |
|  |  | SA2 | Identify and utilize appropriate sources of information to analyze significant public health issues, applying data to reach defensible conclusions. | EPA 13, 14 |
|  |  | SA3 | Accurately describe the organization and basic financial models of the UAE’s health care systems and the potential impact of every system on patients for whom the student has provided care. | EPA 13 |
|  |  | SA4 | Accept and report personal biases and errors, identify potential sources of errors, and develop action plans to reduce the risk of future errors. | EPA 7 |
|  |  | SA5 | Collaborate with stakeholders inside and outside the healthcare system to coordinate optimal care and improve health. | EPA 7, 13 |
|  |  | SA6 | Apply knowledge of health advocacy, systems, and policy to identifying strategies for reducing health disparities and promoting individual and population health. | EPA 13, 14 |
|  |  | SA 7 | Demonstrate accountability to patients, society, and the profession by responding to societal expectations of physicians. | EPA 7 |
|  |  | SA8 | Demonstrate a commitment to patient safety and quality improvement. | EPA 7 |
|  |  | SA9 | Demonstrate commitment to equitable distribution of healthcare resources to under-privileged strata of the population of UAE by providing cost-  effective and quality health care. | EPA 13, 14 |
|  |  | SA10 | Demonstrate commitment to cater to the unique demands of the floating  population and predominantly expatriate demographics of UAE. | EPA 13, 14 |

**Annex 2**

# List of Skills and Clinical Presentation

## **List of Skills**

This chapter includes essential skills the medical graduate should acquire. Skills are classified into five categories.

##### Basic Medical and General Aspects of Practical Skills:

- 1. Taking all necessary steps to prevent infection spread before, during, and after patient care.
  2. Use of personal protective measures (using gloves, gowns, and masks).
  3. Sterilization of equipment and solutions preparation.
  4. Safe disposal of clinical waste.
  5. Correct techniques for handling and moving patients including patient lifting and handling objects or people in the clinical care context using methods that help avoid injury to patients, oneself, or colleagues.

##### Communication and Intellectual Skills:

- 1. Applying a consultation framework.
  2. Establishing & maintaining rapport with patients.
  3. Interviewing (history taking, information gathering).
  4. Imparting information to patients:
     1. Shared decision-making
     2. Disclosure, counseling, and patient education
     3. Getting an informed consent
     4. Breaking bad news
     5. Truth telling (admitting errors & mistakes)
  5. Communicating in writing:
     1. Writing patient's records
     2. Ordering investigations
     3. Prescribing
     4. Writing referral notes
     5. Writing discharge notes
     6. Certifying death
  6. Communicating electronically.
  7. Self-assessment and peer assessment.
  8. Effective communication with colleagues.

##### Clinical Examination and Assessment Skills:

##### General Examination Skills

- - 1. Taking vital signs: cardiac/radial pulse, arterial blood pressure, respiration rate, and body temperature.
    2. Measuring height, weight, head circumference and evaluating on a percentile scale.
    3. Calculating and evaluating Body Mass Index (BMI).
    4. General physical examination techniques including inspection, palpation, percussion, auscultation.

##### Systemic Examination Skills

- - 1. Abdominal examination.
    2. Anterior rhinoscopy.
    3. Breast examination.
    4. Cardiovascular examination.
    5. Examination of lymphatic system.
    6. Examination of mouth and throat.
    7. Examination of thyroid gland.
    8. Genitalia examination.
    9. Gynecological examination, including speculum examination.
    10. Hearing tests.
    11. Mental examination.
    12. Musculoskeletal examination.
    13. Neck examination.
    14. Neurological examination.
    15. Ophthalmoscopic examination.
    16. Otoscopic examination.
    17. Performing peripheral vascular examination.
    18. Preparing peripheral blood smear.
    19. Prostate examination.
    20. Rectal examination.
    21. Respiratory examination.
    22. Upper and lower extremities examination.

##### Assessment Skills

- - 1. Antenatal assessment.
    2. Post-natal assessment.
    3. Following growth and development in children.
    4. Differentiating normal and abnormal ECG.
    5. Identifying the areas and techniques of radiographs.
    6. Assessing chest radiographs.
    7. Assessing skeletal radiographs.
    8. Assessing plain abdominal radiographs.
    9. Assessing visual fields.
    10. Assessing APGAR score.
    11. Assessing infant respiratory distress.
    12. Assessing infant/child dehydration.
    13. Assessing fundal height.
    14. Assessing suicidal risk.
    15. Identifying papilledema.
    16. Identifying focal neurological signs.
    17. Estimating Glasgow Coma Score.
    18. Selecting appropriate laboratory and other diagnostic tests.
    19. Assessing common laboratory results (normal versus pathological).
    20. Planning prevention of communicable diseases in the community.
    21. Nutritional assessment.
    22. Using Snellen’s chart for vision assessment.
    23. Color vision assessment by Ishihara Color Vision Test.
    24. Identifying the cause of death correctly.

##### Diagnostic Procedural Skills:

- 1. Performing arterial puncture for blood gas.
  2. Performing capillary blood sampling.
  3. Performing an electrocardiograph (ECG).
  4. Performing basic respiratory function tests.
  5. Performing eye irrigation.
  6. Irrigating external auditory canal.
  7. Performing removal of corneal foreign body.
  8. Inserting anterior nasal pack.
  9. Advising patients on how to obtain a sample of urine.
  10. Drawing venous blood, venous access.
  11. Testing blood groups.
  12. Collecting a swab.
  13. Collecting samples for occult blood in feces.
  14. Performing pregnancy testing.
  15. Observing lumbar puncture.
  16. Observing peritoneocentesis (ascitic tap).
  17. Performing peak flow measurement.
  18. Performing PAP smear.
  19. Performing PPD.
  20. Using microscope.
  21. Observing bleeding and clotting time.
  22. Urinalysis (by dipstick) and urine microscopic examination.
  23. Measuring blood sugar by glucometer.
  24. Taking samples for cultures (throat, urine, blood, cervix, etc).
  25. Managing blood samples.
  26. Taking blood cultures.

##### Therapeutic Procedural Skills:

- 1. Performing IV injection and administering IV therapy.
  2. Performing IM injection.
  3. Performing intradermal injection.
  4. Performing subcutaneous injection.
  5. Performing trauma emergency including: 5.5.1
  6. Performing primary trauma survey.
  7. Applying cervical collar.
  8. Performing volume resuscitation (including blood transfusion).
  9. Performing handling of unconscious patient.
  10. Applying plaster & immobilizing joints.
  11. Performing enema.
  12. Performing wound care.
  13. Performing basic burn care.
  14. Performing basic suturing.
  15. Performing incision and drainage of abscess.
  16. Performing first aid.
  17. Performing peripheral puncturing of a patient’s vein.
  18. Observing blood transfusion (preparation for blood transfusion).
  19. Performing bleeding control by pressure and tourniquet.
  20. Performing basic restraint for extremities, elastic bandage.
  21. Performing stabilizing and restraining neck and spine.
  22. Recognizing and relieving an obstructed airway.
  23. Performing basic cardiac life support.
  24. Performing cleaning foreign body, placing airway, Heimlich maneuver.
  25. Observing defibrillation.
  26. Observing endotracheal intubation.
  27. Observing tracheostomy & chest tube insertion.
  28. Performing nasogastric tube insertion.
  29. Performing gastric lavage.
  30. Performing bladder catheterization (male and female).
  31. Performing normal vaginal delivery.
  32. Performing assisted vaginal delivery.
  33. Fabricate drugs for preparing medicine forms that suit intravenous parenteral administration injection.
  34. Performing dosage calculation and medication administration.
  35. Showing rational prescribing skills.
  36. Calculating the correct units of insulin and use of the sliding scales a patient requires, the strength of insulin solution to be used, and how to be used.
  37. Instructing patients on the correct use of inhalers.
  38. Performing nebulizer treatment.
  39. Using of local anesthetics.
  40. Performing appropriate aftercare and appropriately after procedure.
  41. Providing guidance for and follow-up of contraception practices.
  42. Performing guidance for breastfeeding.
  43. Planning nutrition according to age.
  44. Immunization assessment: advice and decision-making.

Annex 3

**Mapping of EmiratesMEDs EPAs to Relevant Clinical Skills**

| **EPAs** | **Relevant clinical skills** |
| --- | --- |
| EPA 1: Obtain, perform, and document a focused or comprehensive history and physical examination that identifies relevant clinical findings. | 1.1, 1.2, 2.1, 2.2, 2.3, 2.5.1, 3.1.1, 3.1.2, 3.1.3, 3.1.4, 3.2.1, 3.2.2, 3.2.3, 3.2.4, 3.2.5, 3.2.6, 3.2.7, 3.2.8, 3.2.9, 3.2.10, 3.2.11, 3.2.12, 3.2.13, 3.2.14, 3.2.15, 3.2.16, 3.2.17, 3.2.19, 3.2.20, 3.2.21, 3.2.22, 3.3.1, 3.3.2, 3.3.3, 3.3.9, 3.3.10, 3.3.11, 3.3.12, 3.3.13, 3.3.14, 3.3.15, 3.3.16, 3.3.17, 3.3.21, 3.3.22, 3.3.23 |
| EPA 2: Synthesize information from a clinical encounter to generate and prioritize a justified differential diagnosis. | 2.1, 2.3, 2.8, 3.1.1, 3.1.4, 3.2.1, 3.2.4, 3.2.11, 3.2.14, 3.2.21, 3.3.4, 3.3.5, 3.3.6, 3.3.7, 3.3.8, 3.3.14, 3.3.15, 3.3.16, 3.3.17, 3.3.18, 3.3.19 |
| EPA 3: Select, request, and interpret common diagnostic and screening tests to answer clinical questions and inform patient care. | 2.5.2, 3.3.4, 3.3.5, 3.3.6, 3.3.7, 3.3.8, 3.3.18, 3.3.19, 3.3.22, 3.3.23, 4.1, 4.2, 4.3, 4.4, 4.9, 4.10, 4.11, 4.12, 4.13, 4.14, 4.17, 4.18, 4.19, 4.20, 4.22, 4.23, 4.24, 4.25, 4.26 |
| EPA 4: Formulate, communicate, and implement a patient-centered management plan, including follow-up and escalation when needed. | 2.4.1, 2.4.2, 2.5.3, 2.5.4, 2.5.5, 2.6, 2.8, 3.3.18, 3.3.19, 5.1, 5.2, 5.3, 5.4, 5.8, 5.17, 5.34, 5.35, 5.36, 5.37, 5.38, 5.40, 5.41, 5.42, 5.43, 5.44 |
| EPA 5: Accurately document a clinical encounter in the patient record, including relevant findings, assessment, and plan. | 2.5.1, 2.5.2, 2.5.3, 2.5.4, 2.5.5, 2.6 |
| EPA 6: Deliver a concise, organized oral presentation of a clinical encounter that includes pertinent findings, clinical reasoning, and management plan. | 2.1, 2.3, 2.8, 3.3.18, 3.3.19 |
| EPA 7: Formulate answerable clinical questions, retrieve and appraise relevant evidence, and apply it to advance patient care. | 2.6, 2.7, 3.3.18, 3.3.19, 5.35, 5.44 |
| EPA 8: Give and receive patient handovers using a structured approach that ensures safe transfer of responsibility and accountability for care. | 2.1, 2.5.1, 2.5.4, 2.5.5, 2.6, 2.8 |
| EPA 9: Collaborate effectively with members of an interprofessional team to develop, communicate, and document a shared patient care plan. | 2.4.1, 2.5.1, 2.6, 2.7, 2.8 |
| EPA 10: Recognize patients and situations requiring urgent or emergent care, initiate immediate evaluation and management, and escalate appropriately. | 1.1, 1.2, 1.5, 2.8, 3.1.1, 3.3.10, 3.3.11, 3.3.12, 3.3.14, 3.3.16, 3.3.17, 4.1, 4.3, 4.10, 4.17, 4.23, 4.24, 4.25, 4.26, 5.5.1, 5.6, 5.7, 5.8, 5.9, 5.10, 5.12, 5.13, 5.16, 5.18, 5.19, 5.20, 5.21, 5.22, 5.23, 5.24, 5.25, 5.26, 5.27, 5.28, 5.29, 5.30, 5.38 |
| EPA 11: Obtain and document informed consent for tests or procedures by explaining indications, benefits, risks, alternatives, and confirming patient understanding. | 2.2, 2.4.1, 2.4.2, 2.4.3, 2.4.4, 2.5.1, 2.6 |
| EPA 12: Safely perform general physician procedures appropriate to the local context, with appropriate preparation, technique, aftercare, and documentation. | 1.1, 1.2, 1.3, 1.4, 1.5, 2.4.3, 2.5.1, 4.1, 4.2, 4.3, 4.4, 4.5, 4.6, 4.7, 4.8, 4.10, 4.12, 4.14, 4.17, 4.18, 4.19, 4.22, 4.23, 4.24, 4.25, 4.26, 5.1, 5.2, 5.3, 5.4, 5.10, 5.11, 5.12, 5.13, 5.14, 5.15, 5.17, 5.28, 5.29, 5.30, 5.31, 5.32, 5.39, 5.40 |
| EPA 13: Identify system failures, report patient safety concerns, and contribute to quality improvement actions that promote a culture of safety. | 1.1, 1.2, 1.3, 1.4, 1.5, 2.4.5, 2.6, 2.7, 2.8, 3.3.20, 3.3.24 |
| EPA 14: Educate patients and families and promote the health of the community through counseling, prevention, and health promotion activities. | 2.2, 2.4.1, 2.4.2, 2.6, 3.3.3, 3.3.20, 3.3.21, 5.37, 5.41, 5.42, 5.43, 5.44 |

Annex 4

## **Clinical Presentation**

This section includes most of the common and important clinical presentations the medical graduates should be oriented with. It is subdivided into lists presenting all the systems of the human body within which related common clinical presentations are alphabetically arranged.

##### Musculoskeletal System

- - - Ankle and foot pain
    - Back pain
    - Bone pain/tenderness
    - Buttock, hip, and thigh pain
    - Calf pain
    - Coccydynia (pain in the coccyx)
    - Foot deformities
    - Foot pain/foot ulcers
    - Fracture
    - Hand deformities
    - Joint deformities
    - Joint displacement
    - Joint pain/tenderness
    - Joint stiffness
    - Leg swelling
    - Muscle weakness
    - Muscular pain/tenderness
    - Neck pain
    - Paralysis & paresis
    - Popliteal swellings
    - Shoulder pain
    - Swollen joints

##### Respiratory system

- - - Abnormal breathing sounds
    - Abnormal breathing/labored breathing
    - Apnea
    - Chest pain
    - Cough
    - Daytime sleepiness
    - Hemoptysis
    - Wheeze

##### Cardiovascular system

- - - Altered heart sound
    - Chest pain
    - Cyanosis
    - Dyspnea/Orthopnea
    - Dysrhythmias
    - Edema
    - Hypertension
    - Hypotension
    - Palpitation
    - Parasternal heave & thrill
    - Xanthelasma

##### Genito-Urinary System

- - - Ambiguous genitalia
    - Disturbances of micturition – frequency, polyuria, anuria, oliguria, dribbling, incontinence, urgency
    - Dysmenorrhea
    - Dysuria
    - Empty scrotum
    - Erectile dysfunction
    - Genital lumps, ulcers, rashes
    - Haematuria
    - Impotence/loss of libido
    - Infertility
    - Pain – renal, ureteric, urethral/ flank Pain
    - Pelvic pain and dyspareunia
    - Penile congenital anomalies
    - Premature ejaculation
    - Retention of urine
    - Scrotal mass
    - Scrotal pain

##### Nervous System & Mental Health

- - - Abnormal behaviors
    - Abnormal gait
    - Acute confusion status
    - Altered cognitive status
    - Altered consciousness
    - Anxiety
    - Delusion and thought disorders
    - Depressed mood
    - Disturbed sensation
    - Dizziness, vertigo, and lightheadedness
    - Faints
    - Fits
    - Hallucination
    - Headache
    - Hemiplegia
    - Illusion
    - Insomnia
    - Memory loss
    - Neuropathic pain
    - Personality problems
    - Phobia
    - Tremor and other abnormal movements

##### Paediatric Growth & Development

- - - Abnormal Changes in stature
    - Abnormal development
    - Child abuse
    - Failure to thrive
    - Well-child and anticipatory care

##### GI System

- - - Abdominal pain
    - Abdominal swelling
    - Abnormal tongue appearance
    - Anorectal pain
    - Anorectal swelling
    - Ascites
    - Changes in appetite
    - Constipation
    - Diarrhea
    - Dyspepsia
    - Dysphagia
    - Fecal incontinence
    - Gynecomastia
    - Halitosis
    - Heartburn
    - Hematemesis
    - Hepatomegaly
    - Jaundice
    - Melena
    - Nausea and vomiting
    - Rectal bleeding
    - Splenomegaly

##### Ophthalmology

- - - Diplopia
    - Dry eye
    - Excessive tearing
    - Eye discharge
    - Eye pain
    - Eye twitch
    - Eyelid swelling
    - Leukocoria
    - Nystagmus
    - Ptosis
    - Pupillary problems
    - Red eye
    - Squint
    - Visual disturbances

##### Endocrine System

- - - Delayed or Precocious puberty
    - Gynecomastia
    - Impotence
    - Loss or absence of libido
    - Polydipsia
    - Polyuria
    - Protrusion of eyes
    - Short stature & tall stature
    - Tiredness/General weakness

##### Dermatology

- - - Bruising
    - Clubbing
    - Hair abnormalities
    - Itching
    - Lip ulcers/Lip pigmentations
    - Nail changes
    - Pallor
    - Pigmentation disorder
    - Redness of skin
    - Skin rashes
    - Skin ulcers
    - Soft tissue swellings
    - Swelling of skin
    - Wounds

##### Womens’ Health

- - - Abnormal fundal height during pregnancy
    - Abnormal vaginal bleeding
    - Abuse: physical, psychological & sexual
    - Breast complaints: pain, lumps, and discharge
    - Menstrual disturbances
    - Vaginal discharge and irritation

##### Otolaryngology

- - - Ear discharge
    - Ear pain
    - Epistaxis
    - Facial swelling
    - Hearing disturbances/Deafness
    - Hoarseness/Voice disorders
    - Nasal discharge
    - Neck swelling
    - Oral ulcers
    - Sneezing
    - Snoring
    - Sore throat
    - Speech difficulties
    - Stridor
    - Tinnitus

##### Miscellaneous

- - - Abnormal weight change
